# Preliminary molecular characterization of a proinflammatory and nociceptive molecule from the *Echinometra lucunter* spines extracts

**DOI:** 10.1186/s40409-017-0133-8

**Published:** 2017-10-03

**Authors:** Juliana Mozer Sciani, Bianca Zychar, Luis Roberto Gonçalves, Renata Giorgi, Thiago Nogueira, Daniel Carvalho Pimenta

**Affiliations:** 1 Laboratory of Biochemistry and Biophysics, Butantan Institute, Av. Vital Brasil, 1500, São Paulo, Butantã, SP CEP 05503-900 Brazil; 2 Laboratory of Pathophysiology, Butantan Institute, São Paulo, Butantã, SP Brazil

**Keywords:** Toxins, Sea urchin, *Echinometra lucunter*, Spines, Inflammation

## Abstract

**Background:**

Sea urchins are animals commonly found on the Brazilian shoreline, being *Echinometra lucunter* the most abundant species. Accidents caused by *E. lucunter* have been reported as one of the most frequent in Brazil, and are characterized by intense pain and inflammation, consequence of spine puncture in the skin. In order to characterize such toxic effects, we isolated one molecule that caused inflammatory and nociceptive effects.

**Methods:**

*E. lucunter* specimens were collected without gender distinction. Spines were removed and molecules were extracted, fractionated by RP-HPLC and assayed for inflammatory and nociceptive activity, in a biological-driven fractionation way, until the obtainment of one active molecule and its subsequent analysis by mass spectrometry (MS and MS/MS). For inflammation, intravital microscopy was performed on the mouse cremaster muscle, in order to evaluate rolled, adherent and migrating leukocytes. Paw edema was also evaluated. For the nociceptive activity, the paw pressure test was performed in rats.

**Results:**

One molecule could be isolated and related to the inflammatory and nociceptive activity. Regarding inflammation, increase in adherent and migrating cells was observed in the cremaster muscle after the administration of the molecule. Corroborating the inflammatory response, paw edema was also observed, although only in 20% of controls and 20 min after injection. Additionally, this molecule was able to decrease significantly the pain threshold, characterizing hyperalgesia. This molecule was analyzed by mass spectrometry, and according to the exact molecular mass, isotopic distribution and fragmentation profile, it was possible to propose the molecular formula C_29_H_48_N_3_O_10_.

**Conclusions:**

One isolated molecule from the spine extract of *E. lucunter* is able to elicit inflammation and hypernociception in animal models, which is in agreement with the effects observed in sea urchin accidents.

## Background

*Echinometra lucunter* (Echinodermata: Echinoidea) (Linnaeus, 1758) is the most common and abundant sea urchin species found in Brazil. *E. lucunter* lives in shallow waters, particularly in tide pools and reef slopes [1, 2]. Because of its habitat, it is common that the encounter between bathers and the animal generally leads to accidents, in which mainly the hands and feet of humans are affected by sea urchin spines. This process is due to a mechanism of defense of the animal against the action of the waves [3].

This characteristic makes this urchin species responsible for approximately 50% of the accidents caused by marine animals in Brazil. The symptoms usually surpass trauma and may be pathologically varied. It has been reported that spine penetration causes intense and immediate pain, bleeding, erythema, edema and local myalgia [3–5].

Current treatment includes mainly the spine removal (sometimes by surgery), but also immersion of injury in hot water to inactivate toxins, and topical administration of steroids and antibiotics. Without treatment, acute symptoms may worsen, due to the development of a chronic inflammatory response, associated to the presence of spine fragments and a resultant granuloma formation [6, 7].

The spines are composed of calcium carbonate and are mainly involved in the locomotion and defense of the animal. Sciani et al. [8] reported that *E. lucunter* spines consisted of a porous calcified matrix, symmetrically arranged, with cells rich in secretory granules. Such cells may secrete bioactive compounds, and it was also observed that an ammonium acetate extract (pH 7.3, 24 h) of *E. lucunter* spines is rich in molecules [9]. Such condition would mimic the bioactive/toxic molecule release after the spine punctures human skin. We also have reported that such extract induces acute inflammation and hyperalgesia on mammals, similar to the ones recorded in clinical reports [4, 8, 9]. Nevertheless, to the best of our knowledge, there is no description of toxins from the spines of Brazilian sea urchins.

Moreover, previous analyses by liquid chromatography coupled to mass spectrometry (LC-MS/MS) revealed that only small molecules (below 500 Da) are present in the *E. lucunter* spine extract. This finding differs from the results of other Brazilian sea urchin species, such as *Arbacia lixula* and *Lytechinus variegatus*, which have peptides in addition to small molecules [10].

Considering this, we have looked for the bioactive molecules responsible for the inflammatory and nociceptive effects through biological activity-driven purification strategy (i.e., a biomonitored assay). Establishing relations between molecular entities and biological activities is a crucial step for the better understanding of the participation of urchin toxins in the envenomation process.

## Methods

### Drugs and reagents

All the employed reagents were of analytical grade and were purchased from Sigma Co. (USA), unless otherwise stated.

### Animals

Male Swiss mice (20–25 g) and male Wistar rats (160–180 g) used on the study were treated and maintained under ethical conditions at animal housing facilities of Butantan Institute, Brazil. The present study was approved by the Institutional Animal Care Committee of the Butantan Institute (CEUAIB, protocol number 438/07). All procedures were in accordance with the guidelines for animal experimentation.

### Sea urchin collection and spine extract

Specimens of *E. lucunter* were collected (without distinction of sex, age or size) in São Sebastião, SP, Brazil (23°49′53″S; 45°31′18″W), under license number 13852–1 from the Brazilian Environmental Agency (IBAMA). The spines were removed with scissors, after being anesthetized. The content of the spines were extracted with ammonium acetate (100 mM, pH 7.4) for 24 h, at 4 °C. The extract was processed by solid phase extraction (SPE) using C18 cartridges (Strata®, 55 μm, 70 Å, 5 g/20 mL, Phenomenex Inc., USA) and fraction were eluted with 0, 25, 50, 75 and 100% acetonitrile (ACN), containing 0.1% trifluoroacetic acid (TFA).

### Chromatography

The 25% ACN SPE-fraction was purified by reversed phase high performance liquid chromatography (RP-HPLC) using a binary HPLC system (20A Prominence, Shimadzu Co., Japan). The sample was loaded on a C18 column (ACE C18, 5 μm, 100 Å, 250 mm × 4.6 mm) and the content was eluted by two-solvent system: (A) TFA/H2O (1:1000) and (B) TFA/ACN/H2O (1:900:100) in a 0–80% gradient of solvent B over 20 min, after 5 min isocratic elution with 0% B. The flow rate was constant, set at 1.0 mL.min^−1^ and the oven temperature set at 30 °C. The HPLC column eluates were monitored by a Shimadzu SPD-M20A PDA detector scanning from 200 to 500 nm and the peak of interest was manually collected.

Another chromatographic step was necessary to obtain the high purity molecule. For this step, a carbon-based C18 column was employed (5 μm, 100 Å, 150 mm × 4.6 mm, Hipercarb®, Thermo Scientific, USA), at an isocratic elution of TFA/H2O (1:1000), at constant flow rate of 1 mL.min^−1^, at 4 °C.

After collected, peaks were lyophilized and the dry weight was measured by gravity. Samples were re-suspended in saline solution (0.9%) for biological assays, in a specific concentration for each test (described below).

### Mass spectrometry

Mass spectrometry analyses were performed in an ESI-IT-Tof mass spectrometer (Shimadzu Co., Japan). The sample was diluted in a 50% ACN containing 0.5% formic acid, and was directly introduced in the spectrometer using a Rheodyne 7010 sample loop, at a flow rate of 50 μL.min^−1^, in positive ionization mode. The interface voltage was kept at 4.5 kV, the detector voltage at 1.76 kV and the capillary temperature at 200 °C. The instrument control and data acquisition was conducted by LCMSsolution (Shimadzu Co., Japan), being the mass spectra collected in the 50–2000 *m*/*z* range. For the tandem mass spectrometry (MS/MS) analysis, argon collision energy was kept in 50% and the precursor ions were selected under a 0.5 *m*/*z* window.

In order to deduce the molecular formula of the compound, the mode Formula Predictor (version 1.13) from LCMSsolution was employed, using the following parameters: both configurations (odd or even) of electron ions, only N adducts, actives elements C, H, N, O, and 2-ppm error. The nitrogen rule was either applied or not in the analyses.

### Intravital microscopy of cremaster venules

Leukocyte responses within mouse cremaster venules were assessed by intravital microscopy. Samples (10 μg, diluted in 100 μL of sterile saline) or sterile saline (control) were injected in mice (*n* = 5) randomly selected, into the subcutaneous (s.c.) tissue of the scrotal bag. After 2 h, animals were anesthetized (s.c.) by ketamine (100 mg/kg) and xylazine (10 mg/kg) and the cremaster muscle was exteriorized for microscopic examination in situ as previously described by Baez [11].

During the experimental procedure, mice were maintained on a special board thermostatically controlled at 37 °C, which included a transparent platform on which the cremaster was placed. Leukocyte responses were evaluated by light microscope (Axioplan II, Carl Zeiss, Germany), equipped with Achroplan objectives 10.0/0.25 (longitudinal distance/numeric aperture and 1.60 optovar). Images were captured by a video camera (JVC, Japan) and simultaneously transmitted to a TV monitor. Images were digitalized, converted and analyzed by software (KS 300, Kontron, Carl Zeiss, Germany). One to three post-capillary venules were selected at random. After the stabilization period (initial 10 min), rolling and adhering leukocytes were counted during 3 min in a 100-mm vascular segment. Cells remaining stationary for at least 30 s within a given 100-mm vessel segment were considered firmly adherent leukocytes. Transmigrating leukocytes were also analyzed and quantified as those in the extravascular tissue within 50 mm of each side of the 100-mm vessel segments studied.

### Evaluation of paw edema

Mouse paw edema was induced by intraplantar injection of 10 μg/paw of the sample, diluted in 30 μL of sterile saline (*n* = 5). The contralateral paw received the same volume of sterile saline (control paw). Paw edema was evaluated by a plethysmometer (Letica, Spain) every 10 min, up to 100 min. Results were expressed as the difference (%) of volume between paws injected with sample and sterile saline, and compared to the results before the administration.

### Nociceptive threshold evaluation

Rats (*n* = 8), randomly selected, were evaluated by the paw pressure test before and at different times (1, 2, 4 and 8 h) after intraplantar injection of the sample (10 μg/paw, diluted in 50 μL). The pain threshold was measured using an Ugo Basile® pressure apparatus, essentially as described elsewhere [12]. Briefly, a force of increasing magnitude (16 g/s) was applied to the paw. When the rat reacted by withdrawing the paw, the force (in *g*) required to induce this response represented the pain threshold. The hyperalgesic activity was expressed as the decrease in the force needed to induce the withdrawal response in treated rats compared with control rats that received only sterile saline.

### Statistical analyses

Results related to microcirculation, paw edema and nociceptive evaluation are presented as mean ± standard error of mean. Statistical evaluation of data was carried out by repeated measures two way ANOVA followed by Tukey’s post-test (GraphPad Prism 5, GraphPad Software Inc., USA). Differences of results were considered statistically significant when *p* < 0.05.

## Results

**Fig. 1:**
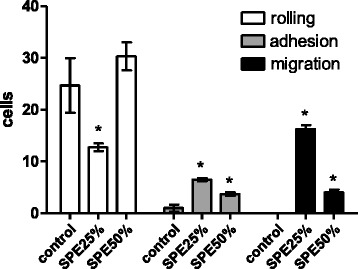
Leukocyte count on the cremaster muscle microcirculation 2 h after 25 and 50% SPE fractions injection. * *p* < 0.05

The spine aqueous extract was initially fractionated by solid phase extraction (SPE), eluted by an acetonitrile step gradient (data not shown). After solvent removal, samples were assayed for inflammatory activity by intravital test on cremaster muscle. As shown in Fig. 1, fractions eluted with 25% and 50% acetonitrile were able to increase the adherent and migrating cells, 2 h after injection when compared to the saline injection, indicating an inflammatory reaction. SPE 25% also caused a reduction of rolling cells. The other SPE fractions (0, 75, and 100% ACN) were not active on these models; therefore, they were not presented.

**Fig. 2:**
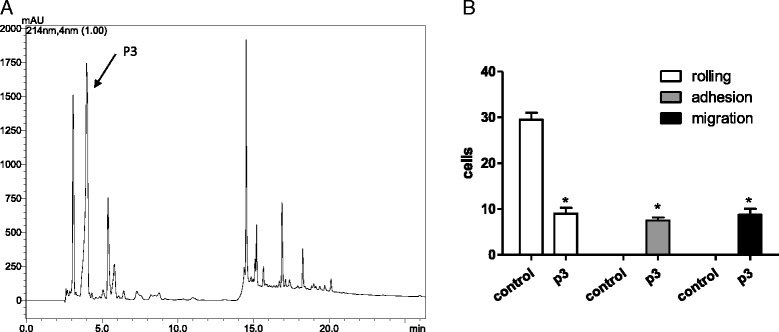
Fractionation of SPE 25% and biological assay of its fractions. **a** RP-HPLC purification of 25% SPE fraction, in a C18 column, elution by 0 to 80% acetonitrile containing 0.1% TFA in water. In the arrow, the proinflammatory peak, named p3. **b** Leukocytes count on the cremaster muscle microcirculation 2 h after p3 injection. * *p* < 0.05

The 25% SPE fraction was selected to be further fractionated by C18-RP-HPLC due to its interesting effect on the increase of migrating cells. The RP-HPLC separation yielded ten peaks that were manually collected, according to the profile shown in Fig. 2a. All the fractions were screened by the intravital assay, and one peak (named p3, arrow in the Fig. 2a) was able to retain the initial inflammatory effect. It caused significant increase of adherent and migrating cells (Fig. 2b), in a similar intensity. The other tested molecules did not cause any effect on the microcirculation.

**Fig. 3:**
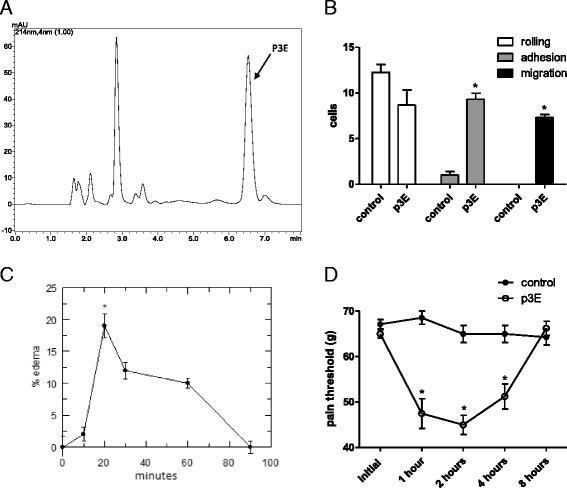
Re-purification of p3 and its inflammatory effects. **a** RP-HPLC purification of p3, in a C18 carbon column, in an isocratic elution with 0.1% TFA in water. The arrow indicates the proinflammatory peak, named p3E. **b** Leukocyte count on the cremaster muscle microcirculation 2 h after p3E injection. **c** Paw edema percentage (control-related) after p3E injection. **d** Pain threshold evaluation after p3E injection. * *p* < 0.05

Complimentary mass spectrometry analysis was performed and it was possible to observe that the fraction was not pure (data not shown). Thus, another chromatographic step was necessary to purify the bioactive molecule. As presented in Fig. 3a, one can observe five new fractions, which were manually collected and assayed on the intravital model once more. Only one molecule (arrow – Fig. 3a, termed p3E) was able to retain the original biological activity (Fig. 3b): the increase of adherent and migrating cells. MS analyses confirmed the purity of the molecule, which was also assayed for paw edema and hyperalgesic activities.

As shown in Fig. 3c, p3E was able to induce only 20% paw edema, 20 min after injection. After 90 min, the edema could no longer be observed. Moreover, p3E was able to decrease significantly the pain threshold, from 1 to 4 h after injection, in comparison to the control group, indicating a clear and intense hyperalgesic activity (Fig. 3d). After 8 h, no effects could be observed anymore.

After successfully determining the biological activities (both inflammatory and nociceptive), a biochemical characterization of the active molecule was performed. For these analyses, MS and MS/MS experiments were conducted in order to measure the exact molecular mass and determine the fragmentation profile, and consequently figure out the molecular formula of the compound.

**Fig. 4:**
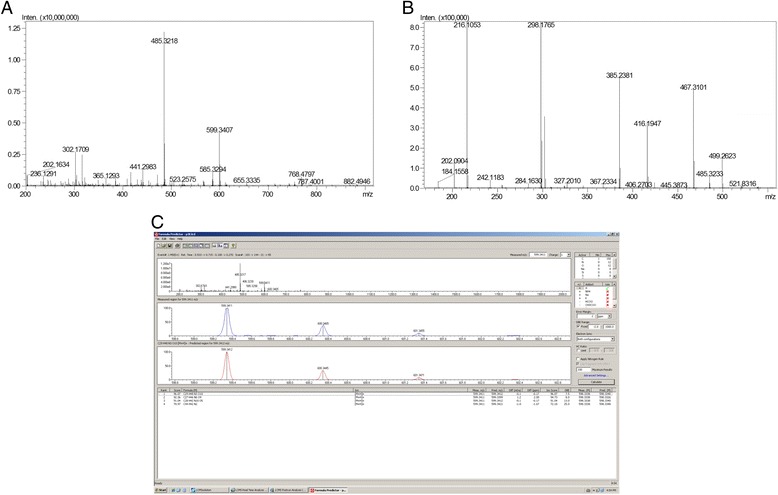
Mass spectrometry analysis of p3E and its characterization. **a** MS profile. **b** Fragmentation of 599 *m/z* ion. **c** Software analysis for prediction of molecular formula

Figure 4a presents p3E MS profile, with two abundant ions: 599.34 and 485.32 *m/z*. The attributed purity of p3E is due to the fact that the fragmentation of 599.34 yields 485.32 (among others), indicating that 485 is a daughter ion of 599, and the 485 observed in the MS spectrum is a spontaneous fragmentation of 599 (Fig. 4b).

The fragmentation pattern of p3E is not typical of peptides, nor de novo sequencing attempts were able to yield a peptide sequence. More likely, 599 is an organic compound. The mass difference between daughter ions indicate the common losses of water, CH_2_ and N.

Then, Shimadzu Formula Predictor Software was used to deduce the molecular formula of the compound, taking into account the exact molecular mass and isotopic distribution pattern, mainly. ‘Active elements’ was set on C, H, N, O, once the isotopic distribution did not indicate the presence of halogens, as Br, I or Cl. The analysis indicates four possible molecular formulas (Fig. 4c), being the score of first very high (96.07) and the error very low (0.17 ppm). Therefore, it was considered the correct molecule: C_29_H_48_N_3_O_10_.

## Discussion

Envenomation by sea urchins has been reported by Brazilian medical facilities to be 50% of all marine accidents. In humans, these accidents are characterized by acute inflammation and pain, and symptoms can evolve to chronic granuloma [13].

Previously, it was believed that these symptoms were caused simply by the penetration of spines in the skin, i.e., a mechanical trauma. However, our group have recently described the proinflammatory and hypernociceptive effects of the aqueous spine extract, i.e., molecules that would be actually released from the spine into the environment (and not a spine macerate or a cell lysate) [9]. Moreover, we have also described the presence of several molecules in such extract, most of which are low molecular mass compounds [10].

In the present work, we have isolated one molecule and, through biological driven fractionation, identified it as being responsible for proinflammatory effects. This molecule was characterized by MS/MS techniques: it is a small molecule (598 Da), whose molecular formula is C_29_H_48_N_3_O_10_. In a search of chemical compounds databases, (1-Dodecyl-1H-1,2,3-triazol-4-yl)methyl 2,3,4-tri-O-acetyl-β-glucopyranoside was found to have the same molecular mass and formula. Although is not a natural product, but a synthetic compound, this molecules and its derivatives caused cytotoxic effects. Thus, the molecule present in the sea urchin can be similar, as well as the biological effects and biochemical characterization [14, 15]. Moreover, this synthetic molecule contains carbohydrates, what explains the low hydrophobicity observed in the chromatography – 25% acetonitrile elution on SPE, 0% B on C18 column and isocratic carbon chromatography at low temperature. Another known compound with same molecular mass was found by Formula Predictor, but ranks at 3rd position score (C_28_H_42_N_10_O_5_): N-(Diaminomethylene)-N′-(L-Lys-L-Lys-L-Phe-)-1H–pyrrole-2,5-dicarboxamide. This molecule and its variants are well characterized.

Up to this moment, several marine natural products have been described, being peptides, terpenoids, alkaloids and polyketides the most frequent ones. Such compounds were mainly obtained from sponges, coelenterates and microorganisms. Echinoderms comprise only 6% of sea organisms reported molecules [16].

For the sea urchins, the isolation and biochemical characterization of (small) molecules is poor and mainly related to secondary metabolites, which participate in the animal’s protection (antimicrobials and antialgals, for example). However, the majority of known molecules were isolated aiming at drug discovery strategies for anti-inflammatory, cytotoxic and antifungal compounds [17].

Among sea urchins, sulfonic acid derivative (from *Brisaster latifrons*), binaphthoquinone and mirabiquinone (from *Scaphechinus mirabilis*), amine salts of sulfated alkenes (from *Temnopleurus hardwickii*), steroidal tri-, tetra-, penta- and hexaoses and aglycone (from *Scaphechinus mirabilis*) have already been identified [17, 18].

Pigments have also been described from several sea urchin species: pyranonaphthazarin, a pigment isolated from the sea urchin *Echinothrix diadema*; echinochrome A, a pentahydroxynaphthoquinone; spinochromes, including echinamines A and B; and a polyhydroxynaphthazarin with a primary amine group [17, 19]. Antioxidant, antimicrobial, antialgal and cardio-protective activities have been related to these pigments [20].

The hedathiosulfonic acids A and B and 6-undecanethiosulfonic acids were isolated from the deep-sea urchin *Echinocardium cordatum*, and exhibited acute toxicity on mice [21, 22].

In this work, we report the isolation and preliminary structural characterization of one small organic molecule, selected by inflammatory effects, characterized mainly by the increase in adherent and migrating cells 2 h after the molecule administration. The kinetics of a leukocyte leaving the blood vessel to the tissue is one major characteristic of an inflammatory process. Such migration is one of the most important physiological events, once it is characterized by the effective in situ leukocytes action, which would culminate in healing the organism through inflammation [23].

Although mild (20%) and fast (20 min), the mouse paw edema – another proinflammatory event – was also observed in this work. Moreover, the edema peak occurred 20 min after injection. Paw edema was also observed in rats, when injected for evaluation of pain threshold (data not shown). The diminishment of the pain threshold observed after p3E administration is in agreement with proinflammatory effects: the intense hyperalgesic activity was observed from 1 to 4 h after molecule administration, which shows an intense and prolonged effect. Only 8 h after p3E administration, the pain threshold returned to basal levels. Thus, data obtained with the edematogenic and hyperalgesic activity suggest that these two phenomena are not correlated.

The decrease in the pain threshold would be a very efficient envenomation strategy, once it would represent a synergic effect between venom molecular features and mechanical trauma derived from the spine skin puncture. In this sense, spines elicit inflammation and toxins (molecules present in the spine) would decrease the pain threshold, leading to a more intense victim reaction. These data is in agreement with the clinical observations, in which patients generally report intense pain and erythema, but no edema is observed.

The aim of this work was to screen and identify at least one molecule responsible for the proinflammatory action, in order to confirm the chemical participation in the clinical manifestations observed. Therefore, a detailed study regarding inflammatory and nociceptive activity, as well as complementary time-course analyses and assessment of the participation of inflammatory mediators, shall be conducted in the future.

We have estimated the minimal number of spines necessary to elicit the inflammatory response in an adult human. In order to achieve this, we have isolated p3E from 60 primary spines (data not shown) which amounted to 200 μg, or approximately 3 μg/spine. Considering that 10 μg is able to cause a local proinflammatory and hyperalgesic effect in mammals, the penetration of only a few spines in the victim, during the envenomation, would be enough to elicit painful local symptoms. It is frequently observed in accidents with humans many spines puncturing the skin, which would explain the intense pain described by the patients. Moreover, we believe that p3E would not be the sole proinflammatory toxin present in *E. lucunter* spines, once other molecules from SPE 25 and SPE 50 also caused inflammation (data not shown), although less intense.

## Conclusion

In conclusion, we have isolated and partially characterized one molecule from *E. lucunter* spines, clearly responsible for inflammatory and nociceptive effects. This finding corroborates our previous published observations that there are indeed toxins in Brazilian sea urchins and that, although not lethal or highly toxic, are truly involved in accidents with sea urchins, adding up to the mechanical effects of spine penetration.

## Abbreviations

ACN: Acetonitrile
LC-MS/MS: Liquid chromatography tandem-mass spectrometry
MS: Mass spectrometry
MS/MS: Tandem mass spectrometry
RP-HPLC: Reverse-phase-high performance liquid chromatography
s.c: Subcutaneous
SPE: Solid phase extraction
TFA: Trifluoroacetic acid
